# Psychological Experiences and Support Needs of Volunteer Psychiatric Interpreters in Japan: A Qualitative Study

**DOI:** 10.7759/cureus.100387

**Published:** 2025-12-30

**Authors:** Ying Zhou, Atsuro Tsutsumi

**Affiliations:** 1 Psychiatry, Faculty of Health Sciences, Institute of Medical, Pharmaceutical and Health Sciences, Kanazawa University, Kanazawa, JPN; 2 Psychiatry, Institute of Transdisciplinary Sciences for Innovation, Kanazawa University, Kanazawa, JPN

**Keywords:** cross-cultural communication, japanese healthcare, medical interpretation, mental health services, psychiatric interpreters, volunteer interpreters

## Abstract

Background: Psychiatric interpreters in Japan-many of whom work on a voluntary basis-play a vital role in bridging critical language barriers for the country’s immigrants while facing unique psychological challenges within a non-certified system.

Aim: This qualitative study aimed to explore the psychological experiences and support needs of volunteer psychiatric interpreters in Japan.

Methods: Semistructured interviews were conducted with 15 medical interpreters recruited via a national dispatch organization (10/15, 66.7%), local networks (3/15, 20%), and hospital referrals (2/15, 13.3%). Data were analyzed using the constant comparative method.

Results: Four major themes emerged: inhibiting factors (e.g., emotional exhaustion, lack of support), facilitating factors (e.g., agency assurance, patient approval), interpreters’ personal qualities (e.g., resilience, altruism), and the pursuit of a professional foundation (e.g., desire for formal training). Participants perceived an emotional toll greater than in general medical interpreting, intensified by Japan’s volunteer-based, under-supported system. None of the participants were full-time psychiatric interpreters, limiting the applicability to highly specialized settings.

Conclusions: Structured psychological training and support networks are recommended to enhance interpreter well-being and improve access to mental health services for immigrants, thereby contributing to psychiatric care, public health outcomes, and cross-cultural communication.

## Introduction

Japan’s non-Japanese speaking population, including 3.77 million immigrants as of 2024 alongside numerous tourists and temporary residents [1], experiences significant language barriers in accessing mental health services [2-4]. A lack of fluency, cultural differences, and limited access to support services can further contribute to psychological distress among migrants in Japan [5]. Although Japan’s healthcare system has made strides in addressing immigrant needs, particularly following the 2020 Tokyo Olympics, significant gaps remain in mental health services due to limited funding for training interpreters [3,6]. A comparative study found that Japanese-Brazilian immigrants residing in Japan exhibited a significantly higher prevalence of probable minor psychiatric disorders (17.8%) compared with their counterparts in Brazil (3.2%) [7]. For example, among Vietnamese migrants in Japan, loneliness caused by language barriers and limited social support has been identified as a significant risk factor for depression and anxiety [8]. These findings align with international guidance that highlights the increased risks of common mental disorders among migrants resulting from language barriers, culture shock, and social isolation and recommends systemic support, including the provision of professional interpreting services and culturally competent care [9].

Language barriers are perhaps the most significant obstacle to mental health care access, highlighting the essential role of trained professional interpreters in clinical settings [6,10,11]. Interpreters in psychiatric settings facilitate linguistic communication while helping practitioners and patients navigate complex cultural and emotional dynamics, serving as cultural mediators and, at times, emotional supporters [12,13]. Fennig and Denov further emphasized their critical role in improving access to care and outcomes for refugees, while also highlighting challenges such as role ambiguity and emotional strain [14].

Language needs in psychiatric care extend beyond surface-level fluency. A multicenter study in Japan found that younger migrants and those with certain diagnoses were more likely to discontinue mental health treatment prematurely, particularly among those who appeared to possess fluent Japanese skills [15]. This finding suggests that hidden language barriers and unmet communication needs may persist even when patients appear linguistically competent, underscoring the necessity of skilled professional interpreters. Their absence or inadequate institutional support not only compromises treatment continuity for patients but also places additional emotional and professional burden on interpreters themselves, further exacerbating risks to their well-being.

Despite their vital role, the psychological experiences of psychiatric interpreters, particularly within Japan’s unique volunteer-driven context, remain underexplored. These interpreters frequently navigate complex emotional interactions without formal training [16,17], placing them at risk of secondary traumatic stress [16,18]. These challenges may include secondary traumatic stress, vicarious trauma, emotional exhaustion, burnout, anxiety, depression, in severe cases-intrusive thoughts or symptom mimicry arising from repeated exposure to patients' distressing narratives [14,16,18,19]. Yin emphasized the overlooked mental health needs of interpreters working with refugees, emphasizing the risks of emotional exhaustion and secondary trauma [19]. Japan’s reliance on a volunteer-based interpreting system, characterized by limited training and institutional support [6], likely exacerbates these challenges, a pattern observed in similar contexts globally [20]. Internationally, studies advocate for structured support, including trauma-informed training and psychological support, to mitigate these burdens [14,19]. This study addresses a critical gap by focusing on the under-explored psychological experiences of volunteer psychiatric interpreters in Japan's unique, noncertified, volunteer-driven system. This study, therefore, explores the psychological experiences and support needs of medical interpreters working in Japan’s psychiatric settings. Adopting a social constructivist framework, the study investigates how interpreters’ emotional realities are shaped by the cultural and institutional contexts of Japan’s volunteer-based mental healthcare system [21].

## Materials and methods

Design

This study employed a qualitative design grounded in a social constructivist framework, which posits that individuals’ experiences are shaped by their social and cultural contexts [21]. This approach was selected to explore the subjective psychological experiences of volunteer psychiatric interpreters in Japan’s noncertified system [14,16,20].

Participants

Fifteen volunteer psychiatric interpreters (11 female, 11 Japanese) were recruited via national dispatch organizations (10/15, 66.7%), local community networks (3/15, 20%), and hospital referrals (2/15, 13.3%). The inclusion criteria required participants to have interpreted in at least two psychiatric sessions and to have at least 3 years of medical interpreting experience. All participants provided written informed consent prior to inclusion. Although the criterion of at least two psychiatric interpreting sessions may appear minimal, it was set to facilitate recruitment, given the limited number of specialized psychiatric interpreters in Japan, while ensuring all participants had direct experience in psychiatric settings. Participants were aged 37-76 (mean: 57.73), with 3-35 years of medical interpreting experience (mean: 14.67). A semistructured interview guide was developed specifically for this study. Prior to obtaining informed consent, participants were informed of the study’s purpose, the interview contents, and ethical considerations.

Data collection

Semistructured interviews were conducted between October 15, 2022, and May 31, 2023. There are a few psychiatric interpreters in Japan, and they are scattered across various regions, usually in urban areas. Considering factors like avoiding the risk of COVID-19, accessibility, and obtaining the maximally geographically varied sample, the interviews were conducted online via Zoom (https://zoom.us), lasting 45-90 min. The research and probing questions (Table 1) were developed based on prior literature [14,16,18], covering psychological experiences, challenges, and support needs. Following the participants’ final approval, all interview recordings were transcribed by the first author into electronic text files. The transcripts were then reviewed by the research team to ensure accuracy before the analytical process.

**Table 1:** Research and Probing Questions

Category	Questions
Research questions	What is the existence, nature, and extent of psychological distress associated with the profession of medical interpreting?
	Are the psychological effects or mental distress experienced by psychiatric interpreters distinct from those of general medical interpreters?
	What are the coping strategies employed by interpreters?
	What are your thoughts on support and supervision systems?
Probing questions	As a medical interpreter (especially a psychiatric interpreter), have you ever had a very difficult time? What that like? Please tell me about it in detail.
	If you have been psychologically affected while working as a medical interpreter (especially as a psychiatric interpreter), what feelings did you experience? What were they and to what degree did you feel them? Have these effects dissipated or persisted? Can you describe the experience in detail?
	After working as a medical interpreter (especially psychiatric interpreting), do you ever feel anxious, depressed, demotivated, uninterested in your next job, or that it has affected your daily life?
	(For those who answered that they feel anxious, etc.) What measures do you usually take to cope?
	Do you feel that working as a medical interpreter (especially as a psychiatric interpreter) has benefited you in any way?
	What kind of support would you like to receive as a medical interpreter (especially a psychiatric interpreter) in the future (e.g., financial, institutional, psychological)?

Interview language and transcription

All interviews were conducted in Japanese, the language preferred by all participants and in which they were fully proficient. The first author, fluent in Japanese, transcribed all interview recordings verbatim into Japanese text files. This approach ensured consistency in language throughout data collection and analysis, preserving the semantic and cultural nuances critical to the social constructivist framework.

Researcher reflexivity

The first author (Z.Y.), a bilingual (Chinese-Japanese) postdoctoral researcher with extensive experience as a conference interpreter and background in cross-cultural health sciences, conducted all interviews. As a non-native Japanese speaker and immigrant researcher in Japan, the interviewer was aware of potential influences of positionality on participant rapport, power dynamics, and data co-construction. Throughout data collection and analysis, ongoing reflection through regular discussions with the corresponding author and research team was employed to identify and mitigate personal assumptions and biases.

Data analysis

The qualitative data were analyzed using the constant comparative method, which is rooted in grounded theory [22]. This analysis followed a four-step process. First, all transcripts were read thoroughly to grasp their overall meaning. Second, semantic units were identified and coded to capture interpreters’ personal qualities, emotional responses, experiences, perceptions, and coping strategies. Third, the codes were compared and grouped into themes through iterative refinement. Fourth, the themes were examined in relation to one another to describe the phenomena revealed by the data. NVivo software was used for coding and data management (https://lumivero.com/products/nvivo/).

Two researchers coded the transcripts independently, generating initial codes such as “emotional exhaustion” and “patient trust.” Discrepancies were resolved through discussions between the researchers, and the coding structure was collaboratively refined. Data saturation was reached when no new codes or themes emerged from the final interviews [23]. Four core themes were identified: inhibiting factors, facilitating factors, interpreters’ personal qualities, and the pursuit of a professional foundation.

Rigor

To ensure the rigor and trustworthiness of this study, the data interpretation and analysis process was supervised by a university professor specializing in qualitative research and psychiatric nursing [24]. In addition, member checking was performed following the generation of the preliminary findings [24]. The results were then shared with selected participants, who confirmed that the findings accurately reflected their experiences and the experiences of their peers.

Ethical considerations

This study was reviewed and approved by the Ethical Review Committee for Research at the researchers’ home university (No. 2022-31, approved October 6, 2022). The explanatory document, which was approved by the Medical Ethics Review Committee, was distributed to the research participants in advance, and sufficient detail was given both in writing and orally. Written informed consent was obtained from all participants prior to their participation. The study was conducted in accordance with the Declaration of Helsinki [25].

## Results

**Figure 1:**
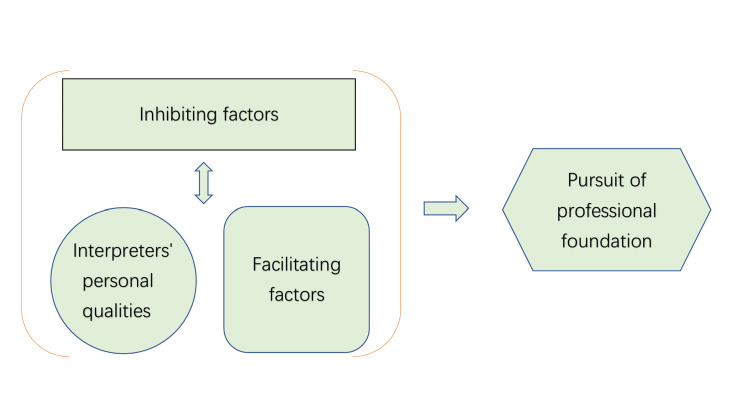
Factor model for medical interpretation experience in Japan Note: This model illustrates the antagonistic relationship between inhibiting factors and the combined facilitating factors/personal qualities that support interpreter resilience and balance. Inhibiting factors (e.g., emotional exhaustion, difficult experiences) act as burdens, while facilitating factors (e.g., satisfaction from patient appreciation and observed impact) and personal qualities (e.g., passion, coping strategies) counteract these challenges, enabling interpreters to maintain psychological equilibrium. This dynamic ultimately contributes to the pursuit of a professional foundation in Japan’s noncertified, volunteer-driven context.

A total of 15 participants were interviewed (Table 2). One participant served as a coordinator for other medical interpreters. Her role involved accompanying medical interpreters to ensure safety. She was deemed suitable for the study due to her partial proficiency in Chinese, her broad experience across a variety of medical scenarios, and her familiarity with the working conditions of medical interpreters. Four main themes were identified in relation to the psychological experiences of psychiatric interpreters: (1) inhibiting factors, (2) facilitating factors, (3) interpreters’ personal qualities, and (4) the pursuit of a professional foundation (Table 3). Facilitating factors and interpreters’ personal qualities were in an antagonistic relationship with inhibiting factors. Inhibiting factors, such as challenging circumstances, were a type of burden, whereas interpreters’ personal qualities, such as passion and a spirit of service, and facilitating factors, such as a sense of fulfillment, served as the foundation to support the balance (Figure 1).

**Table 2:** Demographics of the participants Note: The sample was predominantly female (11/15, 73.3%) and Japanese (11/15, 73.3%), with interpreting experience ranging from 3 to 35 years (mean = 14.7 years). Longer-tenured interpreters predominated. Certain participant letters (K and N) were omitted to preserve anonymity in accordance with ethical guidelines.

Code	Age	Gender	Religion	Nationality	Native language	Languages interpreted	Experience (years)	Area	Employer	Previous job(s)	Primary occupation(s)
A	63	Female	Buddhism	Japanese	Japanese	Portuguese	19	Kantou	NPO	Elementary school teacher	Translator, community interpreter
B	62	Female	None	Japanese	Chinese	Chinese	19	Kantou	NPO	Office worker	Telephone consultant, training instructor
C	57	Female	None	Japanese	Japanese	English	22	Kantou	NPO		Manager, conference interpreter
D	72	Female	None	Japanese	Japanese	English	8	Hokkaido	NPO	University staff, English teacher	Housewife
E	63	Female	Buddhism	Japanese	Japanese	＊	13	Hokkaido	NPO		Coordinator, interpreter accompanying recorder
F	76	Female	None	Japanese	Japanese	English, Spanish	13	Hokkaido	NPO		Pharmacist
G	48	Male	None	Chinese	Chinese	Chinese	6	Hokkaido	Hospital		Hospital staff
H	66	Female	Buddhism	Japanese	Japanese	English	7	Hokkaido	NPO		Medical translator
I	43	Female	None	Japanese	Japanese	Chinese, Korean	3	Hokkaido	Hospital	Nurse	Hospital staff
J	37	Male	None	Chinese	Chinese	Chinese	7	Kantou	Hospital	Assistant professor	Hospital staff
L	44	Male	Buddhism	Malaysian	Chinese	Chinese, Malay, English	8	Kansai	NPO	Office worker	Interpreter
M	64	Female	Christianity	Brazilian	Portuguese	Portuguese, Spanish, French	35	Kansai	NPO	Doctor (in Brazil)	University lecturer, interpreter
O	57	Female	None	Japanese	Japanese	English	15	Kansai	Freelance		Hospital contract interpreter, freelance interpreter, translator
P	50	Female	None	Japanese	Chinese	Chinese	12	Kansai	Freelance		Hospital contract interpreter, freelance interpreter
Q	64	Male	Buddhism	Japanese	Japanese	English, Malay, Indonesian	33	Hokuriku	Hospital	Physical therapist	Japanese language teacher

**Table 3:** Themes and subthemes Note: Emotional exhaustion (subtheme 1c) was the most frequently reported challenge across participants. The four main themes reflect a balance between inhibiting factors and supportive elements (facilitating factors and personal qualities).

No.	Theme	No.	Subtheme	Description
1	Inhibiting factors	1a	Constraints	Obstacles stemming from a lack of social recognition, institutional protection, and adequate support for interpreters.
		1b	Difficult experiences	Encounters with complex and distressing situations such as patient violence, death, and intense suffering.
		1c	Emotional exhaustion	The experience of negative emotions, mental fatigue, and psychological distress as a result of the interpreting work.
2	Facilitating factors	2a	Assurance	Feeling protected and supported by the structures and innovations of the dispatch agency or hospital.
		2b	Approval	Receiving recognition and validation of one's value from patients and healthcare professionals.
		2c	Satisfaction	The sense of fulfillment and positive emotions derived from helping others and witnessing patient recovery.
3	Interpreters’ personal qualities	3a	Coping and adaptation	Self-developed strategies and personal resources to protect mental and physical well-being.
		3b	Passion and spirit of service	Intrinsic motivations, including resilience, altruism, and a proactive desire to contribute to society.
4	Pursuit of a professional foundation	4a	Specialty as medical interpreters	The recognition of medical interpreting as a specialized field and the acquisition of corresponding skills and professional boundaries.
		4b	A desire to improve the system	Advocacy for systemic reforms, including better training, support networks, and career development opportunities.

Theme 1: Inhibiting factors

Inhibiting factors are obstacles to the activities of medical interpreters, including social indifference, difficulties experienced, and negative emotions that arise during work. Medical interpreters in Japan are (subtheme 1a) “constrained” by being unrecognized, unprotected, and insufficiently supported. They have (1b) “difficult experiences,” such as with violence and death, causing them to frequently experience (1c) “emotional exhaustion.”

Constraints

Constraints such as a lack of social recognition, protection, and adequate social support for medical interpreters in psychiatry settings restrict the number of individuals able to engage in this work. Psychiatric interpreters operate in an environment that lacks recognition or acknowledgment from the broader public. Concerns have been raised that younger individuals may be discouraged from pursuing this career path due to the insecure nature of medical interpreting jobs and generally low compensation. Medical interpreters often need to work additional jobs to support their livelihoods. Additionally, many hospitals lack a clear plan for the career development of such interpreters.

Despite being a critical part of patient support teams in hospitals, interpreters often receive little to no support when they face challenging situations. Those working at hospitals are often compelled to act alone and may find it difficult to decline work under challenging circumstances. They were also frequently exposed to the risk of infection from COVID-19. Additionally, medical interpretation is not covered by the national health insurance system, making it difficult for many patients to afford the interpreter fee out of pocket. As Participant E said,

We provide our interpretation services for free. We don’t even charge for transportation. When a hospital requests an interpreter, the hospital charges the patient an interpreter fee. However, it is our responsibility to get this fee from the patient, and that is a disadvantage to the patient. So, (hospitals) introduce our association to patients, and we receive requests directly from the patients. In the end, it all came free.

Difficult Experiences

Difficult experiences include the psychiatric interpreter being confronted with complex situations involving violence, death, and painful decisions in a bleak environment. Medical interpreters are sometimes subjected to physical violence and verbal abuse. They witness a miscarriage, stillbirth, the death of a patient, or painful decisions surrounding care. They can also experience pressure and misunderstanding from physicians, be asked by patients for help beyond the scope of their job or capabilities, have their private lives intruded upon, or otherwise face suspicion or rejection.

Sometimes, maintaining professional boundaries with a patient can be difficult, and patients’ attitudes and reactions may vary greatly depending on the interpreter. There are times when interactions do not go well, when certain matters have to be handled outside working hours, or when fights between patients and hospital staff need to be stopped. The interpreters reported encountering unreasonableness, awkwardness, and a lack of consideration. When witnessing or experiencing violence, for which professional psychiatric workers may be more prepared, interpreters were forced to hide their own shock or fear:

When I tried to stop (the patient), he punched me really hard. When I tried to stop him, he just brushed me off with his hand (Participant P). He was saying to me in a one-sided way, like, “You are a thief,” or “You stole something” … or something like that (Participant O).

Emotional Exhaustion

Emotional exhaustion is when a psychiatric interpreter feels negative emotions or has difficulty maintaining a good mental state. Psychiatric interpreters experience a variety of negative emotions that are affected by their work. They also experienced anguish because they had to suppress their emotions due to tension in the work field or because they felt too much empathy for a patient. There was also a high tendency to worry about a patient’s fate. Some interpreters came into the job by chance, and the patients were complete strangers, making a personal connection difficult to establish. Extreme emotional pressure can lead to “burnout” and mental illness, including depression:

It was difficult because the first time I went to a hospital as a psychiatric interpreter, I felt just as sick as the patients, and I sympathized with them so much. Not only could I not eat, but I was so upset that I had to leave early. It was really difficult for me to stay neutral, and sometimes I would listen to them for several hours and feel as if I had the disease myself (Participant O).

Theme 2: Facilitating factors

Facilitating factors include support from others, a sense of recognition, and positive emotions that are positive factors for job performance. Participants reported receiving (2a) “assurance” from the dispatch agency or hospital and also felt (2b) “approval” and (2c) “satisfaction” from patients and medical professionals.

Assurance

Assurance means that an interpreter is protected by a dispatch agency or hospital, which supports the interpreter with its own innovations. Participants reported feeling that private hospitals were proactive in hiring interpreters and that the requirement for professional medical interpreters was recognized and protected by the dispatch agency or hospital. For example, at sites where there was a risk of physical violence or verbal abuse, interpreters were protected from harm by medical personnel. In addition, some dispatch agencies provided a cushioning function by acting as intermediaries, helping participants feel a sense of security as part of a team, including being able to refuse a patient or access support when needed. The work was unstable, but the unit payment was high, so they were somewhat satisfied. In addition, they understood the Mental Health Care Act, which helped them cope with difficult situations. Participant F noted:

The only thing that helps me not to feel dragged down is that in the group, we can talk about anything, based on the principle of confidentiality, so if I go to patient A and another person supports patient A, we share reports via group e-mail. So, I can share my report in the group e-mail, and I also write what I think at that time, so it’s like we can vent within the group, for lack of a better word.

Approval

Approval is the recognition of one’s value as a psychiatric interpreter by patients and healthcare providers. The interpreters were fortunate to be understood by healthcare providers and appreciated by patients for their effective interpreting skills:

When a patient is in trouble and wants to tell the medical staff about it, but is unable to do so, and the patient feels that I was helping him, he will thank me and say, “Thank you so much for today.” I am very happy when they say, “Thank you so much for today” (Participant D).

Satisfaction

Satisfaction refers to the positive experiences that interpreters gain through their work that motivate them to continue working. Participants reported a sense of fulfillment in their work, primarily stemming from the belief that they were helping others. They also experienced positive emotions when witnessing patient recovery. Moreover, they were able to build on past experiences and engage in multicultural and diverse settings. They felt they were able to engage in experiences beyond the scope of their daily lives, developed a sense of unity with patients and medical personnel, and made new friends:

As I mentioned earlier, I have a lot of stress at home, so when I do interpretation, and it goes well, I feel relieved that I did it. It is a very rewarding job (Participant D).

Theme 3: Interpreters’ personal qualities

The qualities of medical interpreters are positive characteristics that a medical interpreter is born with or has developed through life experiences. Participants encountered a range of complex situations but responded with their own strategies for (3a) “coping and adaptation” with (3b) “passion and spirit of service.”

Coping and Adaptation

Coping and adaptation are methods that the interpreters devise on their own to protect themselves physically and mentally. Specific methods include mentally and emotionally preparing in advance, learning to decline request when necessary, developing the ability to switch between tasks smoothly, seeking support through religion or emotional connections, overcoming difficulties by adapting flexibly in the field, and being honest about their feelings. As Participant M said:

I am a Christian, so I always pray. In my work as an interpreter, or in any other work, even before I eat, I always talk with God, word for word, in prayer. Now I have a very sick patient, so I say things like, “Please help me,” “Please stay with me,” and so on.

Passion and a Spirit of Service

Passion and a spirit of service manifests as a strong, tough, and proactive persona that is willing to contribute to society by using one’s knowledge, experience, and skills. Many of the participants were engaged in medical interpreting as a second career. They had a wealth of life experience and possessed multiple valuable qualities. For example, they had a professional identity, positivity, resilience, honesty, mastery, a sense of purpose, curiosity, an eagerness to learn, and the desire to contribute to society. They reported feeling grateful to have a job, an affinity for other cultures, a desire to help others, and compassion for foreigners, which helped them work without expecting remuneration. Moreover, they showed consideration for patients because they believed those patients needed help:

But having a job is a good thing. Whether it is volunteering or not, having something to do is a great way to enrich one’s life. So, I can’t imagine a life where I do nothing every day, or to put it another way, where I live a life of depression with almost no expectations from the world (Participant F).

Theme 4: Pursuit of a professional foundation

The pursuit of a professional foundation refers to the establishment of medical interpretation as a specialty and the drive to improve both oneself and the field. The participants discussed working hard with a volunteer spirit, but realizing that this is not enough, creating a desire to establish their (4a) “specialty as medical interpreters” with (4b) “a desire to improve the system.”

Specialty as Medical Interpreters

Specializing as a medical interpreter involves the acquisition of a professional foundation through patient care and mediation with health care providers. The participants acquired skills in dealing with patients receiving psychiatric care and recognized the differences in their positions from those of medical professionals. They also tried to respond appropriately to different positions depending on their affiliation (e.g., hospital, dispatch agency). They applied the expertise they had acquired in medical interpretation to other similar fields, further solidifying their professional foundation. As they worked, the participants realized that the field of medical interpretation is a specialized area and that maintaining a sense of distance from patients is important:

I think it’s better to create a sense of distance because if the patients are biased all the time, I will not be able to do my job, my main job, so I think it's better to be cautious (Participant J).

A Desire to Improve the System

A desire to improve the system refers to the need for better treatment services, an improved consultation environment, and higher levels of career development. Specifically, the participants expressed a desire to establish a more consistent system, enhance the quality of treatment services, create an environment where they can consult with others, and seek further learning opportunities. Additionally, there was a desire to have a point of contact while in the field and for others to know what they have experienced. Medical interpreting is often perceived as unproductive work, and participants wanted to challenge this perception and improve their working conditions. They aimed to fulfill the core objectives of medical interpretation and provide better service in coordination with healthcare facilities. As Participant L observed,

Personally, I think it is very important to have a network where you can ask for advice. For example, when I work as an interpreter, I am often confronted with medical problems, and it is very important to have an environment where I can ask for help if I have any questions about medical treatment or anything else. For example, the C organization holds a training session on HIV every year, which is very helpful. It is also very important to have an environment where you can consult with other interpreters about cases that you are having trouble with, such as a system to provide feedback to the hospital, and for the hospital to consult with you about how to respond to such cases in the future.

## Discussion

This study illuminated the intricate psychological realities of volunteer psychiatric interpreters in Japan, revealing a dynamic interplay between the inhibiting and facilitating factors that shaped their experiences within a noncertified, volunteer-driven system. The four emergent themes-inhibiting factors, facilitating factors, interpreters’ personal qualities, and the pursuit of a professional foundation align with the social constructivist framework, positing that psychological experiences are co-constructed through social and cultural interactions [21]. The findings underscore an emotional toll perceived by participants as greater than in general medical interpreting, a burden intensified by Japan’s reliance on volunteer interpreters with limited formal training or institutional support [6]. This discussion situates these findings within the broader literature, explores their implications for mental healthcare, and reflects on the cultural and systemic factors unique to Japan.

The inhibiting factors, including emotional exhaustion and lack of social recognition, resonate with international studies highlighting the psychological risks experienced by interpreters in psychiatric settings [14,18,19]. Participants’ accounts of secondary traumatic stress and exposure to violence echo the challenges faced by interpreters globally, particularly when working with vulnerable populations such as refugees [16]. However, Japan’s volunteer-based system introduces unique constraints, such as interpreters bearing financial burdens (e.g., unpaid transportation) and navigating complex patient interactions with inadequate institutional support. This lack of professional infrastructure contrasts sharply with systems in countries such as Australia or the United States, where interpretation work is often formalized with certification and institutional support mechanisms [14]. The social constructivist lens reveals that these systemic deficiencies can shape interpreters’ sense of isolation and devaluation, reinforcing the need for structured support to mitigate emotional strain.

Conversely, facilitating factors (e.g., agency assurance and patient approval) illuminated the resilience and intrinsic motivation sustaining interpreters’ commitment, aligning with research indicating that interpreters in healthcare settings derive a sense of fulfillment from client gratitude, thereby fostering professional commitment despite other challenges [12]. The sense of empowerment from patient recognition enabled participants to navigate high-pressure environments, a pattern observed in studies of interpreters working with vulnerable populations [16]. However, the reliance on personal qualities such as altruism and resilience to counterbalance inhibiting factors raises questions about the sustainability of Japan’s volunteer-based model. This is particularly true for younger or prospective interpreters, who may be deterred by a lack of career prospects or financial instability.

Interpreters’ personal qualities, such as passion and coping strategies, underscored their agency in adapting to Japan’s unique context. Participants’ proactive approaches (e.g., seeking religious support or maintaining professional boundaries) demonstrated adaptive strategies akin to those observed among other interpreters in mental health settings [14]. However, an absence of formal training programs limits the scalability of these individual coping mechanisms. Internationally, trauma-informed training has been promoted as equipping interpreters with tools to manage emotional strain [14]. In Japan, where cultural norms emphasize collectivism and perseverance, interpreters’ reliance on personal resilience may reflect a culturally shaped response while also highlighting a gap in systemic support that could enhance their well-being and effectiveness.

The pursuit of a professional foundation signals the participants’ desire for recognition and systemic reform, a theme resonant with global recommendations for professionalizing interpreting services [9]. The predominance of women in this sample reflects broader trends in Japan’s community interpreting sector-and raises questions about gendered expectations of emotional labor and self-sacrifice in caregiving roles. Participants’ calls for training and support networks reflect a desire to improve their working conditions. In Japan, where mental health stigma and limited funding for immigrant services persist [3], establishing a certified interpreting system could enhance access to care for Japan’s 3.77 million immigrants and address interpreters’ psychological needs. These findings thus indicate a need for policy interventions that balance culture sensitivity with professional standards, ensuring interpreters are equipped to navigate the emotional and cultural complexities of psychiatric settings.

Limitations

This study has provided valuable insights into the psychological experiences of volunteer psychiatric interpreters in Japan. However, several limitations must be acknowledged. First, the sample size of 15 interpreters, while sufficient for qualitative depth and data saturation [23], limits the results’ generalizability to broader populations of medical interpreters or those in other cultural contexts.

The inclusion criteria (at least two psychiatric interpreting sessions and three years of medical interpreting experience; mean = 14.7 years) systematically excluded early-career interpreters. While beneficial for obtaining reflective depth from experienced participants, this exclusion limited insights into onboarding challenges, training gaps, and attrition risks-key concerns for the sustainability of Japan's volunteer interpreting workforce pipeline. The two-session threshold, although necessary for recruitment in this specialized field, may also restrict understanding of long-term or cumulative psychological impacts on interpreters with more extensive psychiatric experience.

Additionally, the predominance of female (11/15, 73.3%) and Japanese (11/15, 73.3%) participants may not have fully captured the diversity of interpreters, particularly the non-Japanese or male interpreters, who may face unique cultural or gender-related dynamics.

The reliance on Zoom for interviews, necessitated by geographic dispersion and COVID-19 precautions, may have also influenced the depth of rapport and emotional disclosure compared with in-person interviews. While the online interviews facilitated access to a geographically varied sample, they may have constrained the exploration of more sensitive topics such as emotional exhaustion. Moreover, virtual settings limit non-verbal cues that are critical to qualitative research [24].

Meanwhile, the study’s focus on volunteer interpreters in a noncertified system was specific to Japan’s context, limiting its applicability to countries with professionalized and/or certified systems. The absence of full-time psychiatric interpreters in the sample further restricted insights into highly specialized settings, with differing emotional and professional demands. Finally, while the social constructivist framework provided a robust lens for understanding the participants’ subjective experiences, it may have deemphasized structural factors (e.g., healthcare policies, economic constraints) that shaped their realities. Future research could integrate mixed methods to quantify the prevalence of emotional exhaustion or explore systemic interventions more comprehensively.

## Conclusions

This study highlighted the complex psychological landscape navigated by volunteer psychiatric interpreters in Japan, revealing the burdens participants face and the resilience they exhibit within the country’s volunteer-driven, noncertified system. The findings underscore the urgent need for systemic reforms to support interpreters’ well-being and enhance access to mental healthcare to address Japan’s growing immigrant population. Structured training programs, trauma-informed support networks, and professional certification are critical for alleviating psychiatric interpreters’ emotional exhaustion and fostering sustainable careers. These interventions would benefit interpreters and improve cross-cultural communication and mental health outcomes for immigrants, aligning with global recommendations for culturally competent care.

Drawing from a social constructivist framework, the study illustrated how interpreters’ experiences are shaped by Japan’s unique cultural and institutional context, where collectivism and volunteerism intersect with systemic gaps in mental healthcare infrastructure. Policymakers and healthcare institutions should prioritize funding for interpreter training and support, acknowledging their dual role as cultural mediators and emotional supporters. Future research should further explore the perspectives of non-Japanese interpreters and younger professionals to ensure inclusivity and address generational shifts in the workforce. Additionally, comparative studies with countries such as Australia (where community interpreting is formalized) could inform strategies to professionalize Japan’s system. This study contributes to the fields of psychology, public health, and cross-cultural care by amplifying the voices of volunteer psychiatric interpreters. By addressing interpreters’ psychological needs and advocating for professional recognition, Japan can shift toward a more equitable, effective mental healthcare system, ensuring that interpreters and the populations receive adequate support in navigating the complexities of psychiatric care.
